# Preoperative Transcatheter Arterial Embolization and Modified Hepatorrhaphy for Severe Liver Trauma: An Emerging Damage-Control Strategy

**DOI:** 10.7759/cureus.86812

**Published:** 2025-06-26

**Authors:** Teppei Tokumaru, Hideaki Kurata, Jin Mitsui, Joji Tomioka

**Affiliations:** 1 Surgery, Kochi Health Sciences Center, Kochi, JPN; 2 Emergency and Critical Care, Yonemori Hospital, Kagoshima, JPN

**Keywords:** absorbable hemostat, damage control, hepatorrhaphy, liver trauma, trauma surgery

## Abstract

The efficacy of hepatorrhaphy in managing severe hepatic trauma remains uncertain. Although perihepatic packing (PHP) is widely employed, it is associated with risks such as infection and abdominal compartment syndrome. These concerns underscore the need for safer and more effective damage-control strategies. We report the case of a 16-year-old female patient who sustained blunt abdominal trauma following a motorcycle collision and presented with hemodynamic instability due to severe hepatic and renal injuries. Preoperative transcatheter arterial embolization (TAE) enabled hepatorrhaphy without the need for PHP. An absorbable oxidized regenerated cellulose hemostat (Surgicel NU-KNIT, Ethicon, Raritan, NJ) was used in place of conventional non-absorbable pledgets. A right nephrectomy was concurrently performed due to extensive hilar avulsion. The postoperative course was uneventful, and no further interventions were required. This case highlights two potential innovations: the integration of preoperative TAE with hepatorrhaphy and the use of an absorbable hemostatic agent. These approaches may enhance hemostatic efficacy and reduce reliance on PHP in selected trauma cases.

## Introduction

Damage-control strategies for severe liver trauma often involve perihepatic packing (PHP) to achieve temporary hemostasis through tamponade [1-3]. However, PHP does not always provide definitive bleeding control and frequently requires early reoperation or adjunctive interventions, such as transcatheter arterial embolization (TAE) [2,3]. PHP is also associated with significant complications, including abdominal compartment syndrome and intra-abdominal infection [3,4].

Hepatorrhaphy is a hemostatic technique used in the management of liver trauma, although its indications remain poorly defined [4,5]. Additionally, the clinical course and treatment outcomes associated with hepatorrhaphy have not been clearly established [4,6]. While absorbable hemostatic agents, such as oxidized regenerated cellulose, are widely employed in elective hepatic surgery [7], their application in trauma settings has not been well characterized [7,8].

Here, we report a case of severe liver trauma managed successfully with preoperative TAE and hepatorrhaphy using an absorbable hemostat. This may represent a feasible alternative to conventional approaches, such as PHP, in selected patients.

## Case presentation

A 16-year-old girl sustained blunt abdominal trauma due to a self-inflicted fall and skidded approximately 20 m in a low-power (50 cc) motorcycle accident. She had no comorbidities and was not receiving any medications. On presentation, she had bruises on the abdomen and lower back and reported severe pain.

Upon arrival at the hospital via emergency services, her blood pressure was 109/57 mmHg, heart rate was 134 bpm, respiratory rate was 28 breaths/min, and temperature was 37.0°C. Laboratory tests revealed hemoglobin of 7.7 g/dL, a prothrombin time of 54.3%, a fibrinogen level of 153 mg/dL, a pH of 7.384, a base excess of 2.9, and a lactate level of 1.3 mmol/L. Laboratory parameters are summarized in Table 1.

**Table 1:** Initial laboratory tests upon arrival Results include hemoglobin level, coagulation markers, and acid-base status. Reference ranges are provided for clinical context.

Parameter	Value	Reference range	Unit
Hemoglobin	7.7	13.5-17.5	g/dL
Prothrombin time	54.3	>70	%
Fibrinogen	153	200-400	mg/dL
pH	7.384	7.35-7.45	–
Base excess	2.9	-2 to 2	mmol/L
Lactate	1.3	<2.0	mmol/L

**Figure 1:**
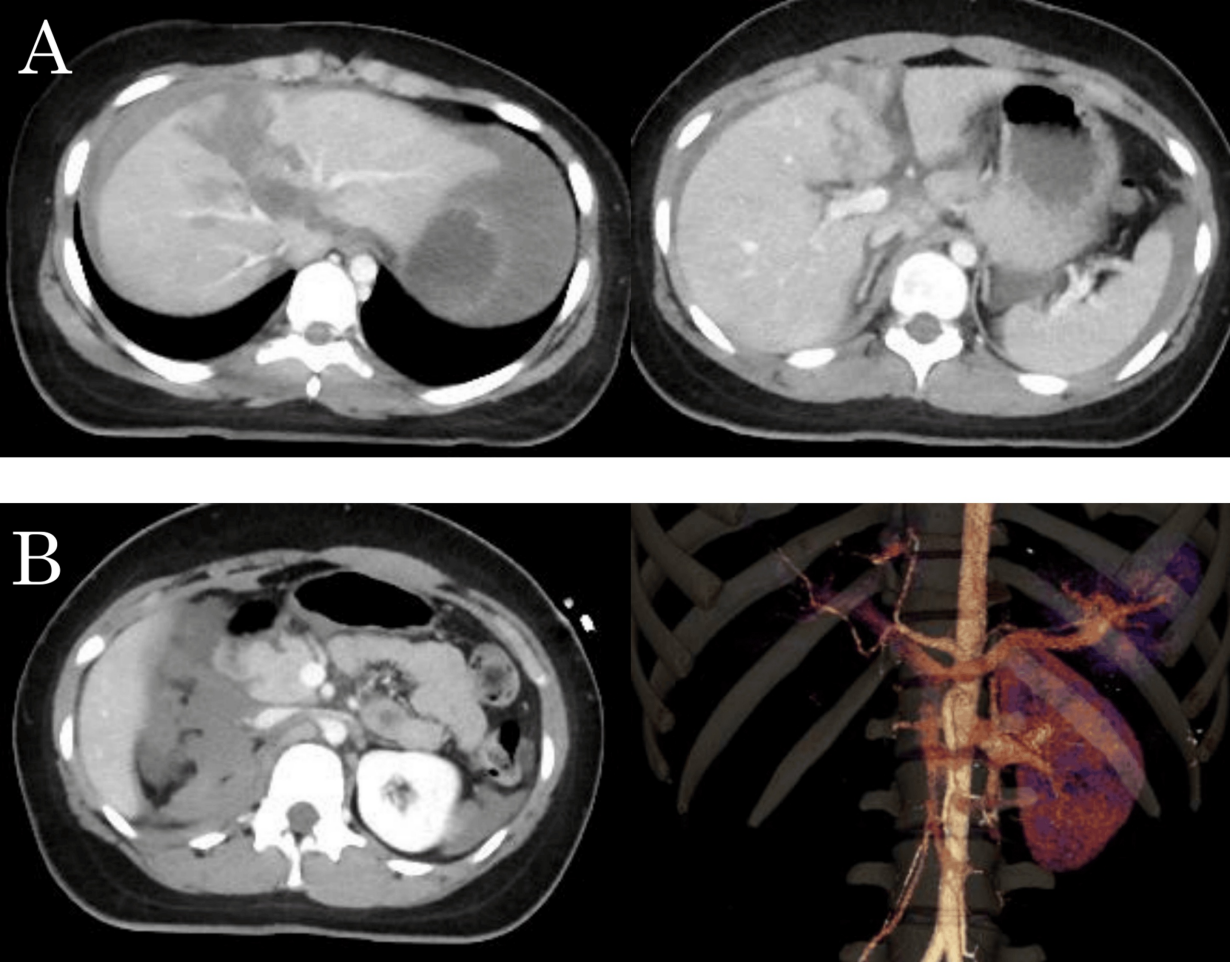
Findings on computed tomography upon arrival (A) Contrast-enhanced computed tomography showing a deep hepatic laceration extending from segment IV to V (AAST-OIS grade IV). (B) Right renal injury with infarction suspected to be a complete hilar avulsion (AAST-OIS grade V). AAST-OIS, American Association for the Surgery of Trauma Organ Injury Scale

Contrast-enhanced computed tomography (CT) showed a 4-cm hepatic laceration involving segment IV with active contrast extravasation, consistent with an American Association for the Surgery of Trauma Organ Injury Scale (AAST-OIS) grade IV liver injury. Additionally, a grade V renal injury was identified due to complete parenchymal infarction of the right kidney, as shown in Figures 1A, 1B.

**Figure 2:**
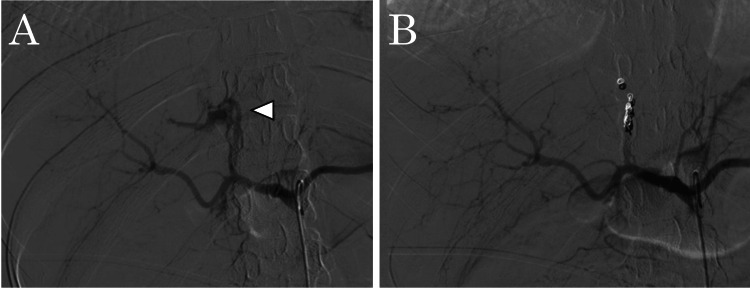
Angiography of hepatic arterial embolization (A) Angiography of the middle hepatic artery demonstrating active contrast extravasation (white arrowhead). (B) The middle hepatic artery was embolized using coils.

Given the hemodynamic instability and extent of anatomical injury, preoperative TAE was performed to stabilize hemodynamics and minimize blood loss both preoperatively and intraoperatively in preparation for urgent laparotomy. Angiography demonstrated active contrast extravasation from the middle hepatic artery, which was promptly embolized using coils, as shown in Figures 2A, 2B.

**Figure 3:**
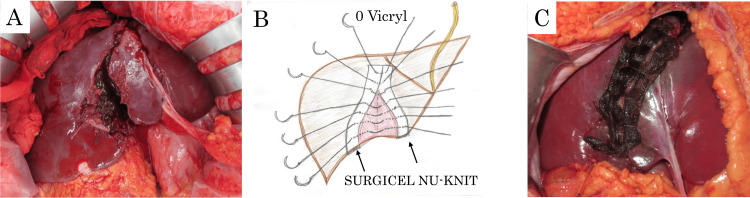
Intraoperative findings and hepatorrhaphy (A) Intraoperative findings showing liver injury with active bleeding controlled by compression. (B) Hepatorrhaphy technique. Absorbable hemostats (Surgicel NU-KNIT) were positioned along the hepatic laceration instead of pledgets, with interrupted sutures using 0-coated Vicryl. This schematic illustration was created by the author based on intraoperative findings. (C) Completion of hepatorrhaphy.

Although PHP was considered, laparotomy revealed ongoing hepatic bleeding that was controlled effectively by direct compression. A tense retroperitoneum, suggestive of renal injury, was also noted. However, there was no evidence of surgical coagulopathy, such as diffuse oozing or impaired clot formation, and the patient did not exhibit hypothermia or acidosis. Therefore, hepatorrhaphy was selected as the damage-control strategy. Following Pringle maneuver occlusion (11 min), hepatorrhaphy was performed using absorbable oxidized regenerated cellulose (Surgicel NU-KNIT, Ethicon, Raritan, NJ) placed along the laceration and secured with simple interrupted 0-coated Vicryl (Ethicon) sutures. Figures 3A-3C show a schematic illustration and intraoperative photographs depicting the liver injury and the hepatorrhaphy technique.

To reinforce the suture line and reduce the risk of foreign body-related complications, an absorbable hemostatic material was used in place of conventional non-absorbable pledgets.

**Figure 4:**
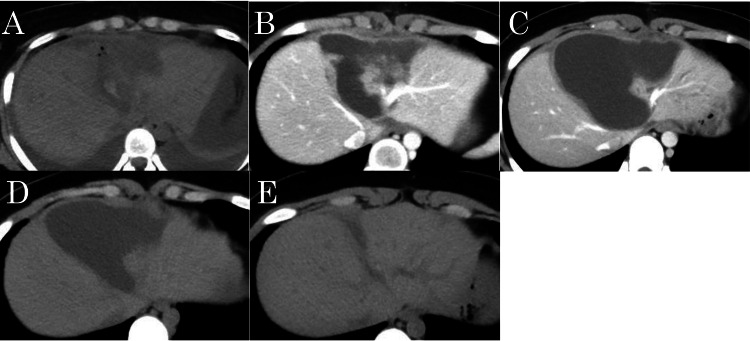
Computed tomography after hepatorrhaphy demonstrating changes over time (A) 7 days; (B) 21 days; (C) 2 months; (D) 3 months; (E) 5 months.

After completion of hepatorrhaphy, the Pringle maneuver was released, and hemostasis was confirmed. As there were no intraoperative signs of surgical coagulopathy, retroperitoneal exploration was undertaken. The right kidney demonstrated near-complete hilar avulsion, deep parenchymal laceration, torsion, and ischemia. On detorsion, active bleeding was observed, indicating that hemorrhage had been temporarily controlled by pedicle torsion or transient tamponade. Given the extent of the injury, right nephrectomy was performed. Following confirmation of hemodynamic stability and overall hemostasis, cholecystectomy and C-tube placement were performed for biliary drainage. The total operative time for laparotomy was 138 min, with estimated preoperative blood loss exceeding 1500 mL. Total transfusion volume from arrival to completion of surgery was 840 mL of packed red blood cells and 960 mL of fresh frozen plasma. No postoperative transfusions were required. On postoperative day 7, cholangiography revealed no bile leakage, and the C-tube was removed. However, localized fluid collection was observed at the site of hepatic injury. Although no bile leakage was evident on cholangiography, the collection was presumed to have resulted from parenchymal infarction and delayed minor bile leakage. The collection resolved spontaneously without intervention. Serial CT imaging demonstrated that the collection reached its maximum size approximately two months after surgery, showed no signs of infection, and gradually decreased in size, nearly resolving by five months postoperatively without intervention, as shown in Figures 4A-4E.

At the three-year follow-up, she remains well without any complications.

## Discussion

This case report emphasizes two key clinical considerations. The first is a damage-control strategy in which preoperative TAE facilitated hepatorrhaphy and single-stage surgery. The second involves a modified hepatorrhaphy technique using an absorbable hemostat instead of conventional pledgets. Both approaches achieved effective hemostasis and resulted in a favorable postoperative course.

The damage-control strategy applied in this patient comprised preoperative TAE followed by single-stage surgery, including hepatorrhaphy. Traditionally, severe liver injuries involving deep anatomical structures and hemodynamic instability have been managed through a staged approach, emergency laparotomy with PHP, and TAE when indicated [1-3]. In this case, due to the extent of the anatomical injury, TAE alone was deemed insufficient, and PHP or hepatorrhaphy was anticipated as potential damage-control interventions [4]. However, intraoperative bleeding was controlled by gentle direct compression, with no persistent oozing observed at the wound edges or dissection planes. Consequently, hepatorrhaphy was performed without PHP, utilizing absorbable hemostats. Right nephrectomy, cholecystectomy, and C-tube placement were conducted concomitantly to achieve single-stage surgery. Similar to PHP, hepatorrhaphy provides compressive hemostasis and is particularly effective for managing venous bleeding [4]. Preoperative TAE reduced hepatic inflow, thereby enhancing the efficacy of hepatorrhaphy and rendering this approach physiologically rational [6]. Avoiding PHP with this strategy is especially significant given the risks associated with PHP, including planned reoperation, abdominal compartment syndrome, and infection [3-5]. PHP, particularly for grade IV or higher liver injuries, often requires postoperative transfusion and is associated with higher complication rates [4,5]. In contrast, our patient required no transfusion, had no infectious complications, and recovered without further intervention, supporting the feasibility of this alternative strategy. To the best of our knowledge, no prior report has described a planned strategy involving TAE followed by hepatorrhaphy. Therefore, we propose this novel approach for managing life-threatening liver trauma.

The modified hepatorrhaphy technique applied in this patient involved the use of an absorbable hemostat (Surgicel NU-KNIT) rather than non-absorbable pledgets. Although non-absorbable pledgets provide mechanical strength, they carry a risk of infection, particularly in abdominal trauma where the surgical field may be contaminated [6]. Absorbable hemostats, such as oxidized regenerated cellulose, are widely utilized and have demonstrated efficacy in elective liver surgery [7,8]; however, reports of their application in trauma settings remain limited [7]. In this case, the absorbable hemostat was effective when employed as a pledget. While postoperative hepatic infarction and minor bile leakage may have occurred, no infection developed, and no additional intervention was necessary. Given the known infection risk associated with non-absorbable pledgets [6], this case suggests that absorbable hemostatic materials may represent a safer alternative in selected high-risk scenarios. This technique may also be applicable as a rescue strategy for critical bleeding in both trauma cases and elective surgeries involving parenchymal organs.

Several additional considerations arise from this case. The decision to proceed with hepatorrhaphy was based on intraoperative findings, notably the absence of persistent oozing or other indications of surgical bleeding tendency [1,4]. Although this approach was adequate, continuous intraoperative monitoring of coagulation parameters, if available, could facilitate more precise surgical decision-making [3]. Alternative hemostatic techniques, such as surface compression with absorbable materials or application of fibrin-based agents, may also be appropriate depending on injury morphology and patient condition [7,8]. Individualized and flexible surgical strategies are essential in trauma care. Furthermore, hemostatic methods widely employed in elective liver surgery may serve as effective damage-control options in selected trauma cases. Additional case accumulation is necessary to further refine and validate these strategies.

## Conclusions

This case demonstrates two key innovations in managing severe hepatic trauma: a damage-control strategy combining preoperative TAE with single-stage hepatorrhaphy and the use of an absorbable hemostat as an alternative to conventional pledgets. In this case, these approaches provided effective hemostasis and potentially avoided PHP, thereby reducing risks such as infection. This strategy may offer a safe and adaptable option for selected trauma cases and could have broader applicability as a surgical rescue technique in elective liver surgery.
